# Local Biological Reactions and Pseudotumor-Like Tissue Formation in relation to Metal Wear in a Murine In Vivo Model

**DOI:** 10.1155/2019/3649838

**Published:** 2019-10-29

**Authors:** Alexander C. Paulus, Kathrin Ebinger, Xiangyun Cheng, Sandra Haßelt, Patrick Weber, J. Philippe Kretzer, Rainer Bader, Sandra Utzschneider

**Affiliations:** ^1^Department of Orthopedic Surgery, Physical Medicine and Rehabilitation, University Hospital of Munich, Ludwig-Maximilians-University, Campus Großhadern, Marchioninistraße 15, 81377 Munich, Germany; ^2^Laboratory of Biomechanics and Implant Research, Clinic for Orthopedics and Trauma Surgery, Heidelberg University Hospital, Schlierbacher Landstrasse 200a, 69118 Heidelberg, Germany; ^3^Biomechanics and Implant Technology Research Laboratory (FORBIOMIT), Department of Orthopaedics, University Medicine Rostock, Doberaner Straße 142, 18057 Rostock, Germany

## Abstract

Metal wear debris and released ions (CoCrMo), which are widely generated in metal-on-metal bearings of hip implants, are also found in patients with metal-on-polyethylene bearings due to the mechanically assisted crevice corrosion of modular taper junctions, including head-neck and neck-stem taper interfaces. The resulting adverse reactions to metal debris and metal ions frequently lead to early arthroplasty revision surgery. National guidelines have since been published where the blood metal ion concentration of patients must consistently be monitored after joint replacement to prevent serious complications from developing after surgery. However, to date, the effect of metal particles and metal ions on local biological reactions is complex and still not understood in detail; the present study sought to elucidate the complex mechanism of metal wear-associated inflammation reactions. The knee joints in 4 groups each consisting of 10 female BALB/c mice received injections with cobalt chrome ions, cobalt chrome particles, and ultra-high-molecular-weight polyethylene (UHMWPE) particles or PBS (control). Seven days after injection, the synovial microcirculation and knee joint diameter were assessed via intravital fluorescence microscopy followed by histological evaluation of the synovial layer. Enlarged knee diameter, enhanced leukocyte to endothelial cell interactions, and an increase in functional capillary density within cobalt chrome particle-treated animals were significantly greater than those in the other treatment groups. Subsequently, pseudotumor-like tissue formations were observed only in the synovial tissue layer of the cobalt chrome particle-treated animals. Therefore, these findings strongly suggest that the cobalt chrome particles and not metal ions are the cause for *in vivo* postsurgery implantation inflammation.

## 1. Introduction

Artificial joint replacement remains the standard procedure for treating the final stage of osteoarthritic joints and is as such one of the most frequently performed surgical procedures in orthopedic surgery [1]. Total hip replacement (THR) has been described as “the operation of the century” with its purpose being to relieve joint pain and reconstruct joint function [2, 3]. However, revision arthroplasty mainly as a result of aseptic loosening still remains a severe complication in patients and a demanding challenge for the surgeons [4]. The pathogenesis of implant-associated aseptic loosening is usually considered to be originated by aseptic inflammatory response, which is mainly initiated by particulate debris and metal ions at the implant-bone interface [5].

Ultra-high-molecular-weight polyethylene (UHMWPE) and cobalt-chromium (CoCr) alloys are commonly used as bearing surface materials in metal-on-polyethylene (MOP) and metal-on-metal (MOM) THR [6]. However, metal wear debris and released ions are continuously generated in MOM bearings of hip implants [7]. Even in MOP bearings, metal wear particles and metal ions are also generated due to the mechanically assisted crevice corrosion of modular taper junctions, including head-neck and neck-stem taper interfaces [8].

Periprosthetic osteolysis with subsequent aseptic loosening still remains a major reason for late period failure after joint arthroplasty [9]. The cause lies in the implant materials used in articulation surfaces and their debris, i.e., the release of abrasive wear particles and their deposition in the periprosthetic tissue [10–12]. On CoCr surfaces, in addition to the mechanical stress, electrochemical and cellular reactions take place on the implant surface, which ultimately lead to corrosion and release of metal ions [13]. The exact molecular mechanism beginning from the formation of the abrasive wear particles to the osteolytic loosening of the endoprosthesis is still not fully understood [14]. One mechanism that has been proposed is that wear particles and ionic corrosion products are initially detected and phagocytosed mainly by the tissue-resident macrophages in the periprosthetic tissue [15]. Then, these wear products can activate macrophages to release an array of proinflammatory cytokines, such as interleukin-1*β*, interleukin-6, and tumor necrosis factor alpha [16], which could activate a wide array of cell types (mainly including lymphocytes, further macrophages, neutrophils, osteoclasts, and fibroblasts) [17]. Therefore, the inflammatory cascade amplified, as well as the balance between osteoclasts and osteoblasts activity is disturbed, which consecutively leads to osteolysis. Usually, what we have observed from revision patients' tissues around the prosthesis are dense aggregates of lymphocytes, thickened synovial layer, and even the formation of the pseudomembrane [18]. Pseudotumor-like tissues were also widely reported in clinical research, which are sterile inflammatory lesions found in the soft tissues surrounding metal-on-polyethylene (MOP) and metal-on-metal (MOM) THR [6].

In light of these discoveries, national guidelines were composed that focus on the metal ion concentration in patients with THR. But to date, it is still unknown why some patients respond differently to others, which lie at the heart of the clinical problem of predicting, preventing, or managing debris or ion-induced reactions. As such, the objective of the present study was to evaluate and compare the biological tissue reactions to metal ions and wear particles in a murine model. The knee joints in 4 groups each consisting of 10 female BALB/c mice received injections with CoCr ions, CoCr particles, UHMWPE particles, or PBS (control). Seven days after injection, the knee joint diameter and synovial microcirculation was assessed via intravital fluorescence microscopy followed by histological evaluation of the synovial layer. The authors hypothesized that significant differences would be found between different experimental groups and the most intense biological reactions would be found in the metal ions treatment group.

## 2. Materials and Methods

### 2.1. Generation of Metal Particles

To generate metallic abrasive wear particles, specimens of a Co29Cr6Mo alloy according to ISO 5832-12/ASTM F1537 were subjected to wear testing using a pin-on-plate simulator. The generated particles showed a mean size in the nanometer range (mean equivalent circular diameter (ECD): 61.25 ± 18.47 nm) with an aspect ratio of 1.69 ± 0.66 and a roundness of 0.64 ± 0.16, The particle shape was thus predominantly oval and round with a small proportion of acicular particles (Table 1). This size and morphology were comparable to those of the clinically produced metal particles in joint revision [19].

### 2.2. Generation of UHMWPE Particles

In order to compare the effect of metal wear particles and polymer-based wear particles on tissue response *in vivo*, commercially available ultra-high-molecular-weight polyethylene (UHMWPE) particles (BioEngineering Solutions Inc., IL, USA) were used. The size and shape of UHMWPE debris are shown in Table 1. The UHMWPE particles were predominantly round, granular, and in the size range of 0.16–26.55 × 10^3^ nm.

The CoCr29Mo6 particle shape was predominantly oval and round with a small proportion of acicular particles (shape: round, 44%; oval, 49%; needle, 7%). UHMWPE particles (GUR 1020) were produced by BioEngineering Solutions Inc., 935N Elmwood Ave, Oak Park, IL 60302, USA.

### 2.3. Generation of Metal Ions

Solid material samples of a Co29Cr6Mo alloy were anodized in a corrosion chamber with an electrical potential against a hydrogen bond electrode to release metal ions. Phosphate-buffered saline (PBS) (Table 2), an isotonic and nontoxic solution [20], was used as the surrounding medium in this study. Meanwhile, PBS, as a commonly used vehicle in intravitreal injection [21] or intra-articular injection [22], is also acceptable to be the vehicle in this study (human extracellular or synovial fluid included all contents of PBS solution). Using high-resolution inductively coupled plasma mass spectrometry (HR-ICP-MS), total metal ion content (cobalt, chromium, and molybdenum) of 20.5 mg/L was determined (Table 3), which was adjusted to the desired target concentration of 200 *μ*g/L using PBS. The target concentration of ions was based on the concentration measured in the synovial fluid of patients with endoprosthesis during revision surgery [23].

### 2.4. Sterilization of the Generated Particles

The metal particles and ions were used to determine their inflammatory activity *in vivo*. To eliminate possible contaminants such as lipopolysaccharides (LPS), or other factors that could influence the inflammatory tissue response, the metal particles were cleaned by an ethanol washing process, whereas the metal ion solution was heat-shocked. The UHMWPE particles were produced LPS-free by washing and sterilization processes by the manufacturer (BioEngineering Solutions Inc., IL, USA). The elimination of endotoxins was determined by using the Limulus amebocyte lysate (LAL) assay (Lonza, Cologne, Germany).

### 2.5. Animal Testing

An animal model, established in the past decades in our institution, was used [5, 24, 25]. 48 female BALB/c mice (Charles River, Sulzbach, Germany), with a mean weight of 23.5 ± 1.9 g, were used in the experiment. The mice were bred for one week at the Walter Brendel Center (LMU, Munich, Germany) prior to injection and were then randomly divided into four groups: PBS control (*n* = 10), UHMWPE particle treatment (*n* = 10), CoCr particle treatment (*n* = 10), and CoCr ion treatment (*n* = 10). The remaining 8 mice were used as reserve animals due to drop out failures (8 cases of extravasation).

All practical experimental steps including animal housing were performed in accordance with the rules and regulations of the Animal Protection Laboratory Animal Regulations (2013) and European Directive 2010/63/EU Act, which is in accordance with the National Animal Protection Law (Protocol number 55.2-1-54-2532-82.12, Government of Bavaria, Germany).

At the day of injection, the particle solutions were sonicated for at least 60 minutes, to prevent agglomeration of particles prior to injection. Under sterile conditions, depending on the group the animal was placed in, 50 *μ*l of each 0.1 vol. % particle suspension or 50 *μ*l of CoCr ions (200 *μ*g/L) solution was injected into the left knee joint using a microcannula (FST, Heidelberg, Germany). 50 *μ*l PBS was used as in the control group. After 7 days, intravital measurements and specimen harvest for histological analysis were performed. The mice were anesthetized by inhalation of 1.2% isoflurane (Fi O_2_ 35%) using a face mask.

### 2.6. Knee Diameter Measurements

In order to see how treatment parameters affected knee joint diameter before and after injection, knee diameter measurements were performed using an electronic digital Vernier caliper (Mitutoyo Deutschland GmbH, Neuss, Germany). The measurement was performed three times per mouse with the average taken as the final result. On the day of particle or ion injection, after animals had been placed under general anesthesia, the first set of measurements were taken 1 h prior to injection, whereas the second measurement was performed 7 days after the injection. Results were then statistically evaluated.

### 2.7. Intravital Microscopy and Microcirculatory Parameters

Using intravital fluorescence microscopy, the synovial microcirculation was evaluated. During the observation, functional capillary density (FCD), rolling leukocytes, and adherent cells were measured as inflammatory reaction indicators [5, 26–29]. FCD refers to the length of the red blood cell-perfused capillaries per unit area, which indicates the quality of capillary perfusion [30, 31]. Rolling leukocytes were defined as white blood cells moving along endothelial cells at velocities lower than the red blood cells in the centerline stream [32]. During leukocyte-endothelial cell interactions, some leukocytes adhere to the blood vessel wall. We defined leukocytes that did adhere to the vessel for longer than 30 seconds as adherent leukocytes.

To measure leukocyte-endothelial cell interaction, the fluorescent marker rhodamine 6G (Sigma-Aldrich, St. Louis, Missouri, USA) was injected intravenously in a single bolus of 0.15 mg/kg^−1^ immediately before the measurement. The FCD was evaluated after intravenous bolus injection of 15 mg/kg^−1^ body weight of the *in vivo* fluorescent plasma marker FITC-dextran (molar mass 150 kDa) (Sigma-Aldrich). Therefore, from the video images, rolling or adherent leukocytes were quantified. Meanwhile, the vessel diameter, RBC velocity, and FCD were recorded according to the FITC images.

### 2.8. Histology

After intravital microscopy, animals were euthanized with an intracardial pentobarbital (Merial GmbH, Hallbergmoos, Germany) injection, and then the left knee of the mice were excised and fixed in 4% paraformaldehyde for 24 h. The joints were then decalcified using 4% EDTA (pH 7.1) for 7 days at room temperature and then washed with PBS. Specimens were processed in ascending grades of ethanol and embedded in paraffin. Sections were then cut sequentially at 3 *μ*m and stained with hematoxylin and eosin. Histological evaluation was performed using the Brackertz scoring system [5, 33]: 0 = normal knee joint; 1 = normal synovium with occasional mononuclear cells; 2 = two or more synovial lining cells and perivascular infiltrates of leukocytes; 3 = marked hyperplasia of synovium and dense infiltration of leukocytes (not only perivascular); 4 = synovitis, pannus formation, and cartilage/subchondral bone erosion.

Data analysis was performed offline using a computer-assisted microcirculation analysis system (Cap-Image, Dr. Zeintl Engineering, Heidelberg, Germany), as established before [5, 27, 29]. Each knee was cut twice, and the thickness of the membrane was evaluated at six defined points in each slice.

### 2.9. Statistics

Statistical evaluation was performed using SPSS Statistics 22 (IBM Deutschland GmbH, Ehningen, Germany). For the statistical analysis, the Kruskal–Wallis test was used to determine statistical variances within groups, followed by a post hoc multiple Mann–Whitney U test. With the aid of the Bonferroni correction, an alpha-error accumulation was neutralized in multiple comparisons. The adjusted level of significance was set at *p* < 0.0083.

## 3. Results

### 3.1. Knee Joint Diameter

**Figure 1:**
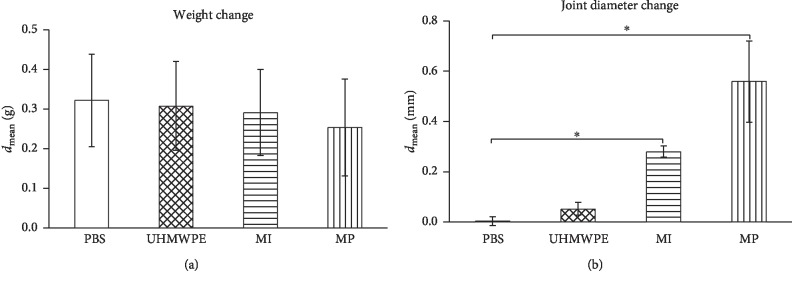
(a) Weight changes of the test animals among all groups in 7 days. (b) The comparison of the knee joint diameter among all groups. PBS, phosphate-buffered saline; UHMWPE, ultra-high-molecular-weight polyethylene; MI, metal ions; MP, metal particles (^*∗*^=*p* < 0.0083).

For 7 days, there were no significant differences (*p*=0.92) in weight changes of the mice among all 4 groups (PBS, MW 0.32 ± SEM 0.12 g; UHMWPE, 0.30 ± 0.11 g; MI, 0.29 ± 0.10 g; MP, 0.25 ± 0.12 g) (Figure 1(a)).

The comparison of the mean values revealed a significant increase in the knee joint diameter after the injection of metal particles (0.55 ± 0.16, *p* < 0.0083) and metal ions (0.28 ± 0.02, *p* < 0.0083) compared to the PBS control group (0.003 ± 0.02) (Figure 1(b)).

After the injection of UHMWPE particles (0.05 ± 0.02, *p*=0.218), a tendency to increase the knee joint diameter could be observed, but not significant with an adjusted value of *p* < 0.0083.

### 3.2. Intravital Microscopy Measurements

**Figure 2:**
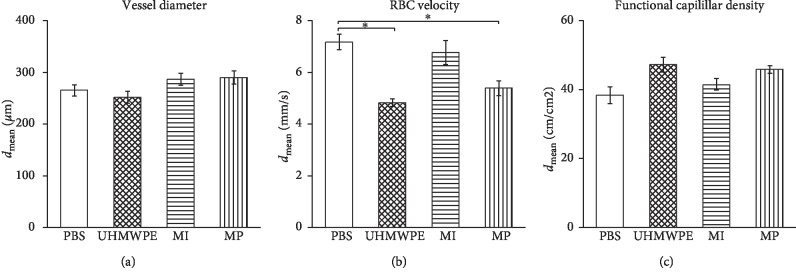
Microcirculatory parameters in all groups. (a) Vessel diameter among all groups. (b) Flow velocity of red blood cells in all groups. (c) The functional capillary density (FCD) showing an increased trend in the MP (*p*=0.023) and UHMWPE (*p*=0.03) groups (^*∗*^=*p* < 0.0083).

There was no significant difference (*p*=0.24) concerning the vessel diameter among all four groups (Figure 2(a)). The test groups injected with UHMWPE (4.82 ± 0.14 mm/s, *p* < 0.0083) or metal particles (5.39 ± 0.29 mm/s, *p* < 0.0083) showed significantly reduced mean RBC flow velocity compared to the PBS control group (7.17 ± 0.30 mm/s). Significance could not be demonstrated comparing the metal ion group (6.78 ± 0.47 mm/s, *p*=0.393) with the PBS control group (Figure 2(b)). In terms of the functional capillary density (FCD), the values of the metal (53.73 ± 1.97, unit: cm/cm^2^; *p*=0.023) and the UHMWPE (45.48 ± 1.06, *p*=0.03) particle groups showed a tendency towards a higher density than those of the control group (35.39 ± 1.35). There was also no significant difference between the CoCr ion group (41.25 ± 1.51) and the control group (*p*=0.247) (Figure 2(c)).

**Figure 3:**
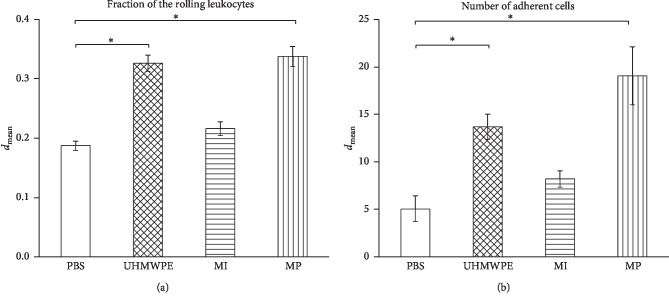
Results of the leukocyte-endothelial cell interaction by intravital microscopy after intra-articular injection of different suspensions. (a) Fraction of rolling leukocytes. (b) Number of adherent cells. Higher numbers of leukocytes were observed in the MP (*p* < 0.0083) and UHMWPE (*p* < 0.0083) groups (^*∗*^=*p* < 0.0083).

For the fraction of the rolling leukocytes, the CoCr particle group (0.34 ± 0.02) and the UHMWPE particle group (0.33 ± 0.013) were highly significantly different compared to the PBS group (*p* < 0.0083). Between the CoCr ions group (0.22 ± 0.012) and the PBS group (0.18 ± 0.01), the parameter did not differ significantly (*p*=0.190) (Figure 3(a)).

The number of adherent leukocytes in the control group was 5.06 ± 1.35. The UHMWPE particle group (13.71 ± 1.98) and the CoCr particle group (19.04 ± 4.41) had a significantly higher number of adherent leukocytes than the control group (*p* < 0.0083). However, there was also no significant difference between the CoCr ion group (8.21 ± 0.84) and the control group (*p*=0.123) (Figure 3(b)).

### 3.3. Histological Results

**Figure 4:**
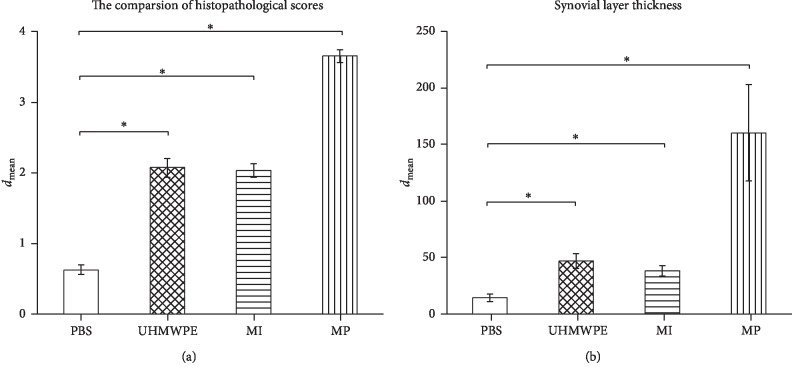
(a) Synovial layer thickness. The synovial membrane measurements revealed a significant increase in thickness across all abrading groups compared to the PBS control group (*p* < 0.0083). (b) The comparison of the histopathological score (^*∗*^=*p* < 0.0083).

On the basis of the histological score in [33], the CoCr particle group (3.65 ± 0.09), the CoCr ion group (2.03 ± 0.10), and the UHMWPE particle group (2.07 ± 0.13) elicited a significantly higher score compared to the control group, whereas the solid CoCr particles provoked the most intensified reaction. The results showed that all three experimental groups had a more severe inflammatory response compared to the control group. Interestingly, there was no statistical difference (*p*=0.481) between the CoCr ion and UHMWPE particle groups (Figure 4(a)).

The membrane thickness (Figure 4(b)) increased massively and statistically significantly (*p* < 0.05) after stimulation with CoCr particles (172.13 ± 40.93 *μ*m) also UHMWPE particles (43.36 ± 6.03 *μ*m) caused significantly (*p* < 0.05) more synovial reactions resulting in a thicker membrane than controls (14.58 ± 3.12 *μ*m). The CoCr particle group showed a severe thickening of the membrane compared to all other groups. This parameter was also significantly different (*p* < 0.05) in the CoCr ion group (38.33 ± 3.91 *μ*m) compared to the control group.

**Figure 5:**
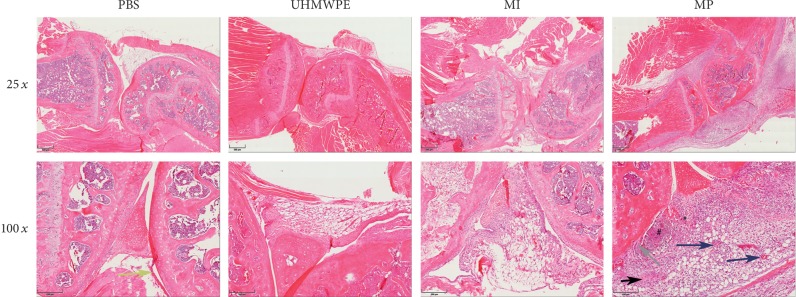
HE staining of sections of the murine knee joints. Histological sections of the MP group showing lymphocytic infiltrates (^*∗*^) in tissue and fibrin exudation (black arrow). Aggregates of metallic wear particles (white arrow) were seen around the necrotic connective tissue (#); capillaries can be found in the obvious inflammatory tissues. Meanwhile, the original synovial membrane (like PBS group, yellow arrow) has been changed. There are some abnormalities of the bone structure between cortical bone and synovial tissue (gray arrow).

Using histological techniques, the mass of soft tissue was found in the MP group, from which the lymphocytic infiltrate (macrophages, eosinophils, giant cells, etc.) and fibrin exudation can be observed. Aggregates of metallic wear particles were seen in the necrotic connective tissue. Capillaries can also be found in the obvious inflammatory tissues. Meanwhile, the original synovial membrane (like PBS group) has been changed. There are some abnormalities of the bone structure between cortical bone and synovial tissue. The inflammatory tissue we found is very close to an inflammatory pseudotumor, so we call it pseudotumor-like tissue. The inflammatory tissue was observed only 7 days after the metal particles were injected into the knee. With the continuous exudation of fibrin and the expansion of the necrotic area, the tissue we found may be consistent with the clinical pseudotumors, all of which are like the foreign body granulomas. These findings could only be seen in the CoCr particle group in 6 out of 10 animals (Figure 5).

## 4. Discussion

CoCr particles instead of CoCr ions showed a considerably increased inflammatory response in all aspects of the study. As known to the authors, this is the first animal model with articular particle stimulation, which was able to prove particle-dependent pseudotumor-like tissue formations.

CoCr alloys have commonly been used for the manufacturing of hip endoprostheses [34]. However, as with any implant material, placed within a closed *in vivo* biological “bioreactor” system, the degradation caused by mechanical, biochemical, and electrochemical processes on the material ultimately lead to its disintegration which sets free molecules, debris, and ions that are not commonly found within a mammalian organism [35]. Some studies have shown that the concentration of CoCr free ions in the blood and urine of postoperative patients is significantly elevated [34], with many of the CoCr metal particles accumulating in the surrounding tissue of the endoprostheses [36]. Researchers have found that UHMWPE particles isolated from *in vivo* studies seem to be mostly globular spheroid particles from 0.1 and 0.4 *μ*m, occasionally with some larger particles up to 250 *μ*m as well as fibrils or shreds [37]. Meanwhile, the median joint fluid levels of CoCr ions in the patients with unilateral arthroplasty is about 201.0 *μ*g/L [23], and CoCr particles retrieved from tissues surrounding prosthesis have been shown to be generally smaller than 50 nm with round or irregular morphologies [38, 39]. But so far, although having known about their morphological parameters, it is uncertain which one can more easily cause aseptic inflammation of the tissue surrounding the prosthesis.

As known to the authors, few relevant studies have assessed the actual biological response of metal particles and metal ions in the pathway of aseptic implant loosening models using actual *in vivo* experiments [24, 27]. Only *in vitro* macrophage culture assays have been used so far to test wear particle effects [40]. As such, it is believed that macrophages are thought to play an important role in particle-induced inflammation, as they regulate the development of other cells especially T-lymphocytes, osteoblasts, and osteoclasts [41]. However, in *in vivo* tissue site, there are a plethora of different tissue layers that have different cells not to mention the vast amount of cytomaterial that migrates through the circulation system. As such, *in vitro* cell culture, whilst beneficial for certain application, cannot reproduce the complex cellular and tissue interactions by true particle-induced immune responses *in vivo*, meaning a reliable and translatable animal model needs to be developed that properly reflects the biological response of abrasive particles and ions in the knee joint. Therefore, our institute has established an animal model to measure the biological response of abrasive particles and ions in the knee joint of mice [5, 28]. The animal models enable us to directly measure the particle-induced inflammation process.

In the present study, this was attempted as the tested animals were exposed to different treatment modalities. By observing the change of knee diameter before and after injection, we found that the difference between the CoCr particles and control groups was statistically significant (*p* < 0.0083). Then, by using intravital fluorescence microscopy, the synovial microcirculation was assessed seven days after injection. Al Saffar et al. [42] demonstrated an increase in angiogenesis in synovial-like tissues surrounding the endoprosthesis and that some up-regulated adhesion molecules emerged that mediate leukocyte-endothelial interactions and inflow of immune competent cells. Leukocyte influx represents a decisive step in the induction of the inflammation based on leukocyte-endothelial cell interactions triggered by adhesion molecules [42]. Inflammatory indicators were measured: the number of adherent leukocytes, functional capillary density, and rolling leukocytes. In terms of these parameters, elevated values in both solid particle groups were found, UHMWPE and metal particles. In contrast, metal ions did not cause significant alterations compared to the control group.

Under the stimulation of various factors, such as inflammatory mediators and the release of free radicals, leukocytes adhere to the vascular wall, so that the interaction time with endothelial cells can be prolonged [43]. White corpuscles that adhere to the capillary wall for longer than 30 seconds are defined as adherent leukocytes [44]. Leukocyte rolling, adhesion, adherent, and chemotaxis are all important steps in the inflammatory response [28, 29]. So, CoCr particles have the strongest inflammatory activity according to adherent leukocytes and the fraction of rolling leukocytes. The inflammatory activity of CoCr ions is significantly lower compared to CoCr particles (*p* < 0.0083).

Pandit et al. [45] performed over 1,300 metal-on-metal total hip replacements. In 17 patients, significant soft tissue mass surrounding the endoprosthesis was found postoperatively. After a series of investigations, it was found that the mass of soft tissue was neither malignant nor infectious. Using histological techniques, B-lymphocytes, T-lymphocytes, and plasma cells were showing a diffuse tissue formation while infiltrating around capillaries. The accumulation of macrophages and eosinophils around the metal particles could also be seen in the soft-tissue mass. According to Pandit et al. [45] who described this formation as a “pseudotumour,” the present study indicates that a similar pseudotumor-like tissue mass had developed at the treated injection site. A common accepted theory of metal wear debris is that it is capable of stimulating the formation of aseptic lymphocytic vasculitis lesions around the prosthesis, which is characterized by diffusing lymphocytic infiltrates and extensive connective tissue necrosis [46]. Various other animal models that have attempted to study aseptic implant loosening, such as hamsters' skinfold-chamber models [46] and “air pouch” models [47], have yielded to date unreliable and limiting information that is not translatable into the clinical scenario, as the particle stimulation site does not correlate with the situation after surgery: the generated wear debris primarily accumulates in the joint that had been replaced. Different from other studies, this study uses an animal model to compare the biological inflammatory response of CoCr particles and CoCr ions in the knee joint *in vivo*. This study found that CoCr particles produced the strongest inflammatory response in the knee joint of mice compared to all other groups, and pseudotumor-like tissue proliferation was frequently observed in the metal particle-stimulated mice which were not reported in literature studies before.

The “inflammatory pseudotumor” contains granuloma-like changes of blood vessels, necrotic tissue, and multinucleated giant cells with the metal debris precipitated [48]. We assume that especially solid metal debris play a key role in the formation of an inflammatory pseudotumor or pseudotumor-like tissue and may have a hypersensitivity reaction to a normal or excess amount of solid metal debris. The histological evaluation by means of staining showed an increased Brackertz score for the metal particle group as well. The metal ion group also differed significantly from the control group. However, why the UHMWPE group's histology seems to be similar to that of the metal ion group in not having developed any form of pseudotumor, despite high ion loads for CoCr, even when higher metal ion concentrations have been shown to trigger the corresponding pseudotumor-like effect, articular and peri-articular issues [49], remains puzzling.

In previous *in vitro* studies, various concentrations of CoCr ions were used to treat various types of cells involved in inflammatory responses, such as monocytes/macrophages, osteoclasts, and osteoblasts. For example, Yang et al. showed that metal ions have a greater inhibitory effect on osteoblast differentiation *in vitro* [50]. Jonitz-Heincke et al. evaluated the effects of CoCr ions on cell differentiation, expression of cytokine, and cell survival in peripheral blood mononuclear cells (PBMCs) and osteoblasts [51]. They found that CoCr ionic solution (200 *μ*g/L) has only marginal effects on human osteoblasts and PBMCs alone; the coculture may provide a comprehensive model to study osteolytic processes in response to CoCr ions [51]. In this study, the CoCr ions group had some inflammatory responses, but no pseudotumor or pseudotumor-like tissue could be induced in the animals despite a high ion load. This might be due to the gradual decrease in the concentration of CoCr ions in and around the knee joint because CoCr ions gradually circulate throughout the body following the circulatory system, thereby gradually attenuating the stimulation effect on the knee, which is not the case for particles.

In general, UHMWPE particles injected in the same dosage as the solid CoCr particles did not show comparable reaction in the murine knee: the Brackertz score of the synovial tissue was less and the intravital microscopy parameters point to a lower inflammatory reaction. Although commercially available UHWMPE particles were used in this experiment, as in fact in clinical realty crosslinked UHWMPE is widely used as PE-bearing material of choice, the results are mostly comparable to a recent study comparing different types of polyethylene [29]: the biological effects on the synovial layer and the subchondral bone of femur and tibia were similar for all the polyethylenes.

There are some limitations to our study. Firstly, the used animal model is an inflammation model and does not allow conclusions to osteolytic processes, which makes the results not comprehensive enough. Secondly, only one concentration of particles and metal ions was used, referring to the national animal laws. In a future study, different particle and ions concentrations will be investigated and the impact of different concentrations on aseptic inflammation. Additionally, the MOM components, especially the first-generation MOM implants, are also commonly fabricated of cast alloys (ASTM F75, ISO 5832-4) [52]. Catelas et al. had demonstrated metal wear particles isolated from various clinically cast or wrought cobalt-base alloys are different. Numerous factors can affect the cellular response to wear particles, including composition, dose, volume, size, and shape of particles [53]. The wrought alloy (ASTM F1537, ISO 5832-12), which we used in this study, may have some limitations for comprehensive understanding of CoCr alloys. Within our continuous study, wear particles and released ions from cast cobalt-base alloy will be tested to see the difference between these two alloys.

*In vivo* corrosion of metal particles going along with a release of small amounts of metal ions is another limitation, as this circumstance could not be addressed and investigated further in this murine model. Measuring ion concentrations in the peripheral blood of the mice might be an indicator for ion release, but only states a systemic ion load, not giving information about the corrosion at the local infiltration site. In fact, this issue is supposed to be addressed in further *in vivo* studies.

Besides, the UHMWPE particles are commercially available and have a different size than *in vivo* particles. This might influence the reaction, as different particles lead to different reactions [29, 54].

## 5. Conclusion

The results of the present study are distinct: CoCr particles produced the strongest inflammatory response in the knee joint of mice. Neither metal ions nor UHWMPE particles showed a comparable biologic reaction. The UHMWPE group and the CoCr ion group indeed had some inflammatory responses. Pseudotumor-like tissue proliferation was frequently observed in the metal particle-exposed mice, but no pseudotumor could be induced in the animals due to high metal ion load.

From the present study, it can be concluded that whilst the inflammatory response by CoCr ions and the UHMWPE particles remains inconclusive probably attributed to the limitation in these materials being applied at a particle and ion concentration inadequate to cause an inflammatory response, CoCr particles seem to be the main cause in local THA surgery-related complications. However, aside from this, our animal model is the first to show the formation of pseudotumor-like structures that are similar to what has been found in periprosthetic tissue after THA and warrants further investigation to elucidate the molecular mechanisms that bring about this unique structure. Indeed, we provocatively can conclude from our results that our design is to date superior to other models tackling this topic by being able to provoke a particle-dependent tissue reaction, and therefore, it is translatable from the *in vivo* to clinical setting.

## Figures and Tables

**Table 1:** Morphological parameters of particles.

Material	ECD (nm)	Aspect ratio	Roundness
CoCr29Mo6 alloy	61.25 ± 18.47	1.69 ± 0.66	0.64 ± 0.16
UHMWPE particles	0.42 ± 0.44 (×10^3^)	1.62 ± 0.78	0.66 ± 0.34

**Table 2:** Chemical composition of PBS solution.

NaCl (g/L)	KCl (g/L)	Na_2_HPO_4_ (g/L)	KH_2_PO_4_ (g/L)
8.1	0.2	1.42	0.2

**Table 3:** CoCr29Mo6 concentrations.

Content (*μ*g/L)	Co	Cr	Mo	Ni
Stock solution	12.0 ± 2.4 (×10^3^)	3.9 ± 0.6 (×10^3^)	0.9 ± 0.1 (×10^3^)	1.3 ± 0.6 (×10^3^)
Experimental solution	120 ± 24	39 ± 5.7	8.8 ± 1.1	12.8 ± 6.0

Total concentration of the CoCr29Mo6 in stock solution as well as in the experimental solution is 200 *μ*g/L.

## Data Availability

The data used to support the findings of this study are available from the corresponding author upon request.
